# Selected Factors Influencing the Intensity of Postoperative Pain in Patients after Orthopedic and Gynecological Surgeries

**DOI:** 10.3390/medicina58111548

**Published:** 2022-10-28

**Authors:** Klaudia Banaś, Paweł Więch, Patrycja Trojnar, Edyta Guty, Mateusz Skórka, Małgorzata Soroń, Anna Nowak, Dariusz Bazaliński

**Affiliations:** 1 Clinical Department of Cardiology with the Acute Coronary Syndromes Subdivision, Clinical Provincial Hospital No. 2 in Rzeszow, 35-301 Rzeszów, Poland; 2 Institute of Health Sciences, College of Medical Sciences, University of Rzeszow, 35-959 Rzeszów, Poland; 3 Department of Nursing, Institute of Health Protection, State University of Applied Sciences in Przemyśl, 37-700 Przemyśl, Poland; 4 St Hedvig Clinical Provincial Hospital No. 2 in Rzeszów, 35-301 Rzeszów, Poland; 5 Center for Forign Language Studies, University of Rzeszów, 35-601 Rzeszów, Poland

**Keywords:** pain, postoperative pain, determinants

## Abstract

Currently, pain is considered the fifth vital sign, and its effective relief is a priority in many surgical departments. The aim of this study was to determine the influence of selected factors on the intensity of postoperative pain after orthopedic and gynecological procedures. The study was conducted in a group of 200 patients undergoing orthopedic and gynecological procedures at the County Hospital in Nisko between August 2017 and January 2018. The method of estimation and document analysis was applied in the course of the study. A standardized tool was used—Polish adaptation of the Melzack Pain Questionnaire (MPQ), an individual documentation of patients and a scientific and research questionnaire developed by the authors. The tests were conducted for three consecutive days from the day of the operation. The strongest pain immediately after surgery was experienced by the patients after general endotracheal anesthesia, and the mildest after full intravenous short-term anesthesia (*p* < 0.05). Procedures lasting more than 60 min were associated with greater pain intensity on all tested days. The sex of the patient did not significantly affect the level of the perceived intensity of postoperative pain (*p* > 0.05). The highest intensity of pain occurred on day 0 after surgery, with a declining trend in the following days (*p* < 0.001). The highest intensity of postoperative pain was caused by gynecological procedures as well as laparotomy and arthroplasty (*p* < 0.001). The variability of the results indicates the need for an individual approach to each patient, both in pain assessment and treatment. Therefore, continuous improvement of the quality of health services provided in accordance with the guidelines for pain relief is necessary.

## 1. Introduction

Currently, pain is referred to “an unpleasant sensory and emotional experience related to actual or potential tissue damage, or similar to that related to actual or potential tissue damage” (Association for the Study of Pain, IASP, 2020) [1]. Numerous studies indicate that postoperative pain is a problem for a large number of patients suffering from severe postoperative pain. Despite various advancements in medicine and pharmacology, postoperative pain, and more precisely its elimination, is still unsatisfactory. Multiple studies conducted worldwide confirm this fact [2,3,4,5,6]. Inappropriate management of postoperative pain delays recovery, prevents the patient from undertaking rehabilitation, induces both physical and mental suffering, disturbs sleep, lowers the quality of life and increases treatment costs [3,4]. The primary responsibility of each therapeutic team is to ensure that the patient experiences the least possible pain, both before and during medical procedures. Everyone, regardless of age, sex, and background, has the right not to suffer pain wherever possible [7]. In 1995, the American Pain Society recognized pain as the fifth vital sign: “Vital signs are taken seriously. If pain was assessed with the same zeal as other vital signs are, it would have a much better chance of being treated properly. We need to train doctors and nurses to treat pain as a vital sign. Quality care means that pain is measured and treated” [8].

Experts around the world are working to improve the services related to postoperative pain management. The American Pain Society has developed recommendations according to which safe and effective treatment of postoperative pain should be based on a care plan tailored to the individual patient’s needs and the type of surgery [9]. Other professional association guidelines, such as those of the American Society of Regional Anesthesia and Pain Medicine [ASRA] and the American Society of Anesthesiologists [ASA], have indicated general recommendations for optimal postoperative pain management [6,9]. The Polish Society for the Study of Pain has introduced guidelines in Poland for the management of postoperative pain, which are to draw attention to the safety of analgesia administered, the necessity of systematic pain monitoring, patient education, appropriate medical documentation, and training of therapeutic teams in the field of pain relief [10,11]. Unfortunately, the problem of pain management and control is multifactorial and concerns the whole of Europe. Approximately 70% of the 240 million postsurgical patients every year suffer from moderate-to-severe pain [12]. A European survey of 746 hospitals indicated that the management of postoperative pain was suboptimal [13]. Another observational study conducted in 11 European countries, identified orthopedic surgery, preoperative chronic pain, and percentage of time in severe pain on first day as significant risk factors [14]. Results presented by Borys et al. suggested that many patients still experience moderate or severe pain in the postoperative period, even though there are guidelines and methods to treat pain after surgical procedures [15]. A better understanding of the pathomechanisms of postoperative pain for short- and long-term outcomes is essential to improve prophylactic and treatment strategies [16].

In this study, we aimed to assess the influence on type of anesthesia, duration and type of the surgical operation and usage of premedication on the intensity of postoperative pain after orthopedic and gynecological procedures. Therefore, we assumed the following hypotheses: the intensity of postoperative pain is the lowest in patients after regional anesthesia, while the highest is after administration of short-term intravenous anesthesia; patients after long-term treatments feel pain of a higher intensity than patients after short and low-intensity treatments; the use of premedication has no effect on the pain intensity immediately after the procedure; the strongest pain sensations occur on the day 0 after the procedure, with a decreasing tendency in the following days; women experience postoperative pain more strongly; and patients after orthopedic procedures describe higher pain intensity.

## 2. Materials and Methods

### 2.1. Ethics Considerations

The study was carried out in compliance with the ethical norms set out in the relevant version of the Declaration of Helsinki (64th WMA General Assembly, Fortaleza, Brazil, October 2013) and in conformity with Polish legal regulations. The study design was approved by the Bioethics Committee at the University of Rzeszów (2018/03/13i).

### 2.2. Study Design

The method of estimation and document analysis was applied in the course of the study. A standardized tool was used—the Polish adaptation of the Melzack Pain Questionnaire (MPQ) developed by Kazimierz Sedlak [17]. The analysis was based on the medical records of patients of PCK County Hospital in Nisko (Podkarpackie Province, Poland). The study was approved by the management of the abovementioned facility. The results were recorded in a scientific and research questionnaire developed by the authors.

### 2.3. Settings and Participants

The study was conducted from August 2017 until January 2018 in a purposefully selected group of 200 patients of PCK County Hospital in Nisko (100 patients from the Department of Orthopedics and 100 patients from the Department of Gynecology on days 0, 1, and 2 after surgery, where day 0 meant the day of surgery). The mean age was 49 ± 15.04 years, half of the respondents were under 51, while the remaining ones were in the 38–62 age-group. The following inclusion criteria were adopted: being an adult, understanding the questions fully and consciously, and hospitalization minimum 3 days after the surgery procedure. Patients with chronic pain, multiple diseases, with limited mental comprehension or refusing participation in this survey were excluded from the study. The characteristics of the study group are presented in Table 1.

### 2.4. Assessments

Dedicated research tools were used to assess whether the intensity of postoperative pain depended on the type of surgery and the anesthesia used, the extent and duration of the operation, and the sex of the patients. The standardized Melzack Pain Questionnaire (MPQ) and the patient’s medical records were used in the study. The obtained results were collected in the scientific and research questionnaire developed by the authors. The first test with the MPQ was carried out within 24 h after the procedure, and the next ones on the following days. The Polish adaptation of the MPQ by Kazimierz Sedlak consists of 74 adjectives arranged in 20 groups, each of which contains from 2 to 6 pain descriptors. There are 4 dimensions: sensory dimension of pain (groups 1–10), emotional dimension of pain (groups 11–15), subjective dimension of pain (group 16), and an additional category containing adjectives assessing various pain characteristics (groups 17–20). Pain assessment is determined by: number of selected words (LWS), pain assessment index (WOB), and current pain intensity (AIB). In LWS, a maximum of 20 points can be scored, and the greater the number of words indicated, the greater the pain intensity. WOB is determined on the basis of the values assigned to individual adjectives in the questionnaire. AIB is a five-point scale (from 0—no pain to 5—unbearable pain) with which the patient determines pain symptoms at the time of the examination. In the additional tables, the subject indicates the symptoms associated with pain, assesses sleep, nutrition, vital activity, and the duration of pain. In a figure showing the human body outline, the location and radiation of pain is marked [17]. The actual studies were preceded by a pilot study among 30 patients who were included in the proper study group.

### 2.5. Statistical Analysis

The IBM SPSS Statistics 20 program was used for the statistical analysis. Differences between variables were verified using the chi-squared test of independence, Mann–Whitney test, Kruskal–Wallis test, Wilcoxon signed-rank test, and Spearman’s rho correlation coefficient. The choice of nonparametric tests was dictated by the lack of normality of the distributions of the variables (verified with the Kolmogorov–Smirnov and Shapiro–Wilk tests) or the lack of equivalence of the groups (verified by the χ^2^ concordance test and the binomial test). *p* values < 0.05 were considered statistically significant.

## 3. Results

### 3.1. Effect of Anesthesia on the Intensity of Postoperative Pain

The intensity of postoperative pain was statistically significantly dependent on the type of anesthesia used. The analysis of MPQ indices showed that the patients experienced greater pain after general endotracheal anesthesia and lower pain after total short-term intravenous anesthesia. This relationship was maintained on individual days after the procedure (Table 2).

### 3.2. Pain and the Duration of the Procedure

It has been shown that the longer the procedure duration, the greater the pain intensity. On days 0 and 1, procedures lasting more than 60 min caused the highest pain symptoms, indicated in all the domains of the MPQ questionnaire. On day 1, patients experienced the lowest pain after short procedures lasting less than 30 min. The intensity of perceived pain showed a downward trend in the following days (Table 3).

### 3.3. Premedication Used and the Intensity of Pain Immediately after the Procedure

Patients who received premedication reported slightly higher pain than patients who did not receive premedication. The correlation of WOB on day 2 and AIB on days 1 and 2 after surgery was approximately significant. The use of premedication had a small effect on pain in patients in the analyzed time interval (*p* > 0.05) (Table 4).

### 3.4. The Intensity of Postoperative Pain in the Days Following the Procedure

The highest pain intensity was on day 0 after the procedure, then it decreased statistically significantly in the following days (*p* <0.001) (Table 5).

### 3.5. Pain and the Sex of the Patients

The sex of the patients did not significantly affect the level of the perceived intensity of postoperative pain (*p* > 0.05) (Table 6).

### 3.6. The Type of Surgery and the Intensity of Postoperative Pain

The type of surgery significantly influenced the perception of pain (*p* < 0.001) (Table 7). On days 0 and 1, the patients showed the highest intensity of pain after gynecological procedures with laparotomy. This tendency changed on day 2, when the highest LWS and WOB indices were achieved by patients after endoprosthesis. On all three days, the subjects experienced the least pain after gynecological operations performed using the vaginal approach. The highest intensity of postoperative pain (AIB index) was caused by gynecological procedures with laparotomy and endoprosthetics.

## 4. Discussion

The conducted study concerned the determination of the influence of selected factors on the intensity of postoperative pain after orthopedic and gynecological procedures. The analysis included the dependence of the pain intensity on the type of procedure performed and the anesthesia used, the extent and duration of the operation, and the sex of the patients. The intensity of pain in the days following the procedure was examined.

The time that elapses after the surgery affects the intensity of postoperative pain. The highest intensity of pain occurs on day 0 and decreases in the following days. There are reports confirming this relationship in the literature. In the research conducted by Sobieralska-Michalak K. et al., the respondents experienced the greatest pain on day 0 and the lowest on day 3 postoperatively [18]. In a Dutch study of 1490 hospitalized surgical patients, 41% of patients reported moderate or severe pain on the day of the surgery, with a decrease to 30%, 19%, 16% and 14% on the 1st, 2nd, 3rd, and 4th day after surgery [19].

In our study, the analysis of the MPQ sheet showed a dependence of pain intensity on the type of anesthesia performed. Patients experienced greater pain after general endotracheal and subarachnoid anesthesia, and lower after short-term intravenous anesthesia. Ulatowska A. et al. included 82 people hospitalized for surgical treatment in the surgical ward. They distinguished two groups: patients after cholecystostomy performed under general anesthesia and patients after removal of varicose veins of the lower limbs with subarachnoid anesthesia. They showed that the type of anesthesia used had a significant impact on the intensity of postoperative pain. Patients, both immediately after the procedure and at 4, 8, and 12 h, showed higher pain scores on the VAS after general anesthesia [20]. Liszka et al. studied the degree of pain intensity after corrective surgery of the hallux valgus. The subjects were treated under general anesthesia, general anesthesia combined with local preemptive anesthesia, and three types of regional anesthesia. Pain measurements using the VAS at 2 and 4 h after surgery were significantly higher after general anesthesia than in other cases [21]. Nakahashi K. et al. examined nearly 10,000 patients who underwent subarachnoid or general anesthesia for elective surgery. They indicated that the most common reasons for patients’ dissatisfaction with care were the use of subarachnoid anesthesia, the most unsatisfactory factor, followed by epidural anesthesia, postoperative pain, vomiting, nausea, and memories of extubation [22]. Grochonas E. et al. conducted a study among 84 surgical patients. They used three pain scales: analogue, numerical and visual. The obtained results did not confirm the influence of the applied anesthesia on the level of perceived pain. Patients anesthetized with general and regional anesthesia assessed pain at a comparable level on the 1st and 3rd days after surgery [23].

It was assumed that the longer the treatment duration, the higher the pain intensity. MPQ values were the highest after procedures lasting more than 60 min, and the lowest after short-term surgery—less than 30 min. The intensity of perceived pain showed a downward trend in the days following the treatments. The results of our study are in line with the study by Mei W. et al. among over 1700 patients. They showed that longer operation time influences the intensity of postoperative pain. Among other factors, the authors also indicated younger age, obesity, the use of nitrous oxide, and location of the procedure within the musculoskeletal system and within the abdomen [24].

The use of premedication before anesthesia had a minor effect on pain in postoperative patients. Patients who received midazolam showed slightly lower pain values after the procedure. The use of gabapentin would be more effective. It was proved by Panah Khahi M. et al. in the study of two comparative groups in which gabapentin and placebo were administered that in the first group, the pain sensation was lower immediately after the procedure than in the second [25]. Alayed A. et al., conducting a systematic review, showed that preventive administration of gabapentin was effective in reducing postoperative pain, the consumption of opioid analgesics, and nausea and vomiting [26]. The use of adjuvants before surgery may be helpful in relieving postoperative pain, but it depends on the formulation used and its mechanism of action.

Our study indicated that the sex of the patients did not significantly affect the level of the perceived intensity of postoperative pain. However, there is no consensus in the literature regarding this statement. Mei W. et al. reported that female sex belongs to the group of independent risk factors for the development of postoperative pain shortly after a procedure [24]. Kołodziej W. and Karpel E., in their studies with the use of the MPQ questionnaire, proved that more pain intensity occurs in women than in men [27]. Similar conclusions in their research on pain intensity after cardiological procedures were presented by Szczudłowski B. and Płaszewska-Żywko L. [28]. Regardless of the type and extent of surgery, women reported slightly higher pain scores in the research of Gerbershagen HJ et al. [29]. Haghighi M.J. et al., in their studies, showed that in patients undergoing general surgery, the mean pain intensity was significantly higher in men than in women [30]. Due to cultural differences between the studied groups, it would be advisable to conduct comparative studies.

The type of the surgery performed significantly influenced the perception of pain. On days 0 and 1, the patients reported the highest intensity of pain after gynecological procedures with laparotomy, while on day 2 after arthroplasty this had diminished. On all three days, the subjects experienced the least pain after vaginal gynecological surgeries. In the scientific literature on the treatment of postoperative pain, it is emphasized that thoracic surgeries and abdominal surgeries are the most painful, while surgeries on integuments and limbs are usually associated with much less pain. The location and extent of the procedure, the degree of tissue damage, the direction of the skin incision, and the use of specific analgesia techniques in the perioperative period significantly affect the degree of pain perception in patients [31]. A prospective cohort study comparing pain in the first day after surgery among more than 50,000 patients was conducted in many hospitals in Germany. Pain sensation was assessed on the 11-point NRS after 179 surgical procedures. Obstetrics and orthopedic procedures turned out to be the most painful [32]. The effectiveness of combating postoperative pain with paracetamol was analyzed among the patients of the Gynecology Clinic of the Teaching Hospital in Lublin. All patients were anesthetized with the same method. Laparoscopic operations, slightly shorter and usually involving less tissue trauma, resulted in much lower pain intensity in the first 12 h after surgery than operations with laparotomy [33].

This study has several limitations that could have influenced the results. First, it was a single-center study. Another limitation concerns the study sample, limited to one population—patients treated surgically in the gynecological and orthopedic wards. It would be worth extending the research to other surgical departments such as general surgery, vascular surgery, or cardiosurgery. Moreover, the small number of participants in the study may be an additional factor limiting the critical interpretation of the obtained results; therefore further studies on larger groups of patients are needed.

## 5. Conclusions

The factors that can influence the perception of postoperative pain are extensive: long-lasting procedures with a significant degree of tissue traumatization, and the use of general endotracheal and subarachnoid anesthesia. Patients report the strongest pain on the day of the procedure, with a decreasing tendency in the following days. The highest intensity of postoperative pain was caused by gynecological procedures as well as laparotomy and arthroplasty. The use of premedication and being a woman or a man do not significantly affect the perception of pain. The variability of the results indicates the need for an individual approach to each patient, both in pain assessment and treatment. Having considered the above, further studies would allow for a thorough analysis of the presented variables.

## Figures and Tables

**Table 1:** Characteristics of the study group.

ParameterN = 200	Department of Orthopedics		Department of Gynecology		Total		*p*-Value
	n	%	n	%	n	%	
**Sex**							**<0.001**
Women	50	50.0%	100	100.0%	150	75.0%	
Men	50	50.0%	0	0.0%	50	25.0%	
**Type of procedure**							**<0.001**
The upper limb and shoulder	36	36.0%	0	0.0%	36	18.0%	
The lower limb	58	58.0%	0	0.0%	58	29.0%	
Hip or knee arthroplasty	6	6.0%	0	0.0%	6	3.0%	
Gynecological surgery with laparotomy	0	0.0%	39	39.0%	39	19.5%	
Transvaginal gynecological surgery	0	0.0%	61	61.0%	61	30.5%	
**Duration of the procedure**							0.407
Less than 30 min	14	14.0%	10	10.0%	24	12.0%	
30–60 min	47	47.0%	56	56.0%	103	51.5%	
More than 60 min	39	39.0%	34	34.0%	73	36.5%	
**Type of anaesthesia**							**<0.001**
Subarachnoid	58	58.0%	58	58.0%	16	58.0%	
Intravenous regional general endotracheal	15	15.0%	0	0.0%	15	7.5%	
Total intravenous short-term	12	12.0%	32	32.0%	44	22.0%	
**Premedication**							0.159
Yes	82	82.0%	89	89.0%	171	85.5%	
No	18	18.0%	11	11.0%	29	14.5	

N—number of subjects; n—number of observations; %—percent.

**Table 2:** Effect of anesthesia on the intensity of postoperative pain.

MPQ Index	Day	Type of Anesthesia								*p*-Value
		Subarachnoid		Intravenous Regional		General Endotracheal		Total Intravenous Short-Term		
		M	SD	M	SD	M	SD	M	SD	
LWS	0	5.26	2.16	5.33	1.50	6.59	2.50	3.88	1.56	**<0.001**
	1	11.89	6.72	13.47	4.76	15.73	7.70	9.04	5.37	**<0.001**
	2	4.00	1.79	3.87	1.30	4.75	2.11	3.00	1.22	**<0.001**
WOB	0	8.58	4.85	8.33	3.6	10.41	5.86	6.72	4.35	**0.014**
	1	2.73	1.31	2.80	0.86	3.09	1.46	1.72	1.06	**<0.001**
	2	5.51	3.20	4.60	2.10	6.05	3.35	3.20	2.53	**<0.001**
AIB	0	2.91	0.82	3.00	0.65	3.39	0.69	2.08	0.86	**0.001**
	1	2.28	0.75	2.13	0.74	2.93	0.76	1.48	0.59	**<0.001**
	2	1.48	0.69	1.53	0.52	1.64	0.69	0.92	0.49	**0.367**

MPQ—Melzack Pain Questionnaire; LWS—number of selected words; WOB—pain assessment index; AIB—current pain intensity; M—mean; SD—standard deviation; *p*—level of statistical significance of differences.

**Table 3:** Pain and the duration of the procedure.

MPQ Index	Day	Duration of the Procedure						*p*-Value
		Less than 30 min		30–60 min		More than 60 min		
		M	SD	M	SD	M	SD	
LWS	0	3.54	0.98	5.35	2.20	6.04	2.32	**<0.001**
	1	2.79	0.93	3.92	1.59	4.59	2.15	**<0.001**
	2	1.71	1.00	2.67	1.22	3.04	1.46	**<0.001**
WOB	0	7.71	3.16	12.66	7.03	13.84	7.08	**<0.001**
	1	5.75	2.72	8.56	4.46	9.95	5.93	**<0.001**
	2	3.04	1.99	5.10	3.14	6.25	3.20	**<0.001**
AIB	0	1.92	0.65	2.96	0.78	3.18	0.82	**<0.001**
	1	1.33	0.48	2.36	0.77	2.56	0.82	**<0.001**
	2	0.83	0.38	1.42	0.63	1.70	0.70	**<0.001**

MPQ—Melzack Pain Questionnaire; LWS—number of selected words; WOB—pain assessment index; AIB—current pain intensity; M—mean; SD—standard deviation; *p*—level of statistical significance of differences.

**Table 4:** The use of premedication and the intensity of pain immediately after the procedure.

MPQ Index	Day	The Use of Premedication				*p*-Value
		Yes		No		
		M	SD	M	SD	
LWS	0	5.50	2.29	4.72	2.02	0.078
	1	4.11	1.88	3.59	1.55	0.194
	2	2.77	1.35	2.24	1.21	0.065
WOB	0	12.63	7.05	11.69	6.23	0.545
	1	8.80	5.05	8.34	5.07	0.586
	2	5.40	3.15	4.52	3.39	0.063
AIB	0	2.96	0.86	2.66	0.90	0.108
	1	2.36	0.84	2.03	0.82	0.051
	2	1.49	0.69	1.21	0.62	0.057

MPQ—Melzack Pain Questionnaire; LWS—number of selected words; WOB—pain assessment index; AIB—current pain intensity; M—mean; SD—standard deviation; *p*—level of statistical significance of differences.

**Table 5:** The intensity of postoperative pain in the days following the procedure.

MPQ Index	Day 0		Day 1		Day 2		*p*-Value
	M	SD	M	SD	M	SD	
LWS	5.39	2.26	4.03	1.84	2.69	1.34	**<0.001**
WOB	12.50	6.93	8.73	5.04	5.27	3.19	**<0.001**
AIB	2.92	0.87	2.31	0.84	1.45	0.69	**<0.001**

MPQ—Melzack Pain Questionnaire; LWS—number of selected words; WOB—pain assessment index; AIB—current pain intensity; M—mean; SD—standard deviation; *p*—level of statistical significance of differences.

**Table 6:** Pain ailments and the sex of patients.

MPQ Index	Day	Sex				*p*-Value
		Woman		Man		
		M	SD	M	SD	
LWS	0	5.37	2.36	5.42	1.96	0.555
	1	4.01	1.98	4.08	1.32	0.201
	2	2.64	1.43	2.84	1.02	0.199
WOB	0	12.28	7.22	13.14	6.01	0.151
	1	8.65	5.33	8.96	4.08	0.145
	2	5.23	3.34	5.40	2.72	0.408
AIB	0	2.90	0.92	2.96	0.70	0.814
	1	2.33	0.88	2.26	0.72	0.645
	2	1.41	0.71	1.56	0.61	0.177

MPQ—Melzack Pain Questionnaire; LWS—number of selected words; WOB—pain assessment index; AIB—current pain intensity; M—mean; SD—standard deviation; *p*—level of statistical significance of differences.

**Table 7:** The type of surgery and the intensity of postoperative pain.

MPQ Index	Day	Type of Procedure										*p*-Value
		The Upper Limb and Shoulder		The Lower Limb		Hip or Knee Arthroplasty		Gynecological Surgery with Laparotomy		Transvaginal Gynecological Surgery		
		M	SD	M	SD	M	SD	M	SD	M	SD	
LWS	0	5.06	1.98	5.50	2.17	6.67	1.37	7.18	2.66	4.20	1.33	**<0.001**
	1	3.75	1.20	4.21	1.68	4.67	1.86	5.36	2.55	3.11	1.07	**<0.001**
	2	2.56	0.94	2.98	1.32	4.17	1.47	3.18	1.59	2.03	1.06	**<0.001**
WOB	0	13.22	7.44	13.21	6.22	13.00	5.48	16.62	8.0	8.70	3.74	**<0.001**
	1	8.78	4.38	9.76	4.64	10.00	7.87	11.10	6.76	6.08	2.55	**<0.001**
	2	4.72	2.46	5.98	3.12	8.83	4.02	6.54	3.81	3.75	2.26	**<0.001**
AIB	0	2.92	0.81	3.05	0.60	3.33	0.52	3.59	0.68	2.31	0.87	**<0.001**
	1	2.08	0.81	2.38	0.67	3.00	0.63	3.03	0.74	1.85	0.73	**<0.001**
	2	1.39	0.49	1.62	0.59	2.17	0.75	1.85	0.78	1.00	0.52	**<0.001**

MPQ—Melzack Pain Questionnaire; LWS—number of selected words; WOB—pain assessment index; AIB—current pain intensity; M—mean; SD—standard deviation; *p*—level of statistical significance of differences.

## Data Availability

The data presented in this study are available on reasonable request from the corresponding author: pwiech@ur.edu.pl.
